# Oxidative Stress and Neuroinflammation Potentiate Each Other to Promote Progression of Dopamine Neurodegeneration

**DOI:** 10.1155/2020/6137521

**Published:** 2020-07-03

**Authors:** Jingyi He, Guofu Zhu, Guoqing Wang, Feng Zhang

**Affiliations:** Joint International Research Laboratory of Ethnomedicine of Ministry of Education and Key Laboratory of Basic Pharmacology of Ministry of Education, Zunyi Medical University, Zunyi, Guizhou, China

## Abstract

Parkinson's disease (PD) is a chronic and complex disease of the central nervous system (CNS). Progressive loss of dopamine (DA) neurons in midbrain substantia nigra is considered to be the main cause of PD. The hallmark of PD pathology is the formation of Lewy bodies and the deposition of *α*-synuclein (*α*-syn). The mechanisms responsible for the progressive feature of DA neurodegeneration are not fully illustrated. Recently, oxidative stress and neuroinflammation have received extensive attention as two important entry points in the pathogenesis of PD. The occurrence of oxidative stress and neuroinflammation is usually derived from external influences or changes in internal environment, such as the accumulation of reactive oxygen species, exposure to a toxic environment, and the transformation of systemic inflammation. However, PD never results from a single independent factor and the simultaneous participation of oxidative stress and neuroinflammation contributed to PD development. Oxidative stress and neuroinflammation could potentiate each other to promote progression of PD. In this review, we briefly summarized the conditions of oxidative stress and neuroinflammation and the crosstalk between oxidative stress and neuroinflammation on the development of PD.

## 1. Introduction

Parkinson's disease (PD) is the second most common and intricate neurodegenerative disease characterized by tremors, rigidity, and bradykinesia. There have been plenty of studies on the pathogenesis of PD, including hereditary factors, oxidative stress, and neuroinflammation, among which nongenetic factors account for the dominating causes. For a long time, the accumulation of *α*-synuclein (*α*-syn), the loss of dopamine (DA) neurons in the substantia nigra, and the activation of glial cells are closely related to PD. Nevertheless, the pathogenesis of PD is quite complicated, and it is difficult to describe accurately with a single factor. Increasing evidence indicates that the pathogenesis of PD might be composed with a series of complex factor interactions, particularly in the two major factors, oxidative stress and neuroinflammation. The synergistic effect of these two factors on DA neurodegeneration needs further study.

Oxidative stress has been identified as a common underlying mechanism for cell dysfunction and eventual cell death. Under normal circumstances, the production of reactive oxygen species (ROS) in the body is in balance with the antioxidant activities of cells, and oxidative stress occurs when this balance is destroyed [1]. Inflammation is a protective condition that repairs and regenerates damaged tissue or cells in the body and removes infectious agents, parasites, or toxins. Similarly, neuroinflammation is initially a protective response in the brain, but excessive neuroinflammation is harmful, in fact, also inhibiting the regeneration of neurons [2]. It has been reported that oxidative stress and neuroinflammation play synergistic roles on the pathogenesis of PD [3]. However, how do these two factors potentiate each other to drive the progression of PD? This review summarized the conditions of oxidative stress and neuroinflammation and crosstalk between oxidative stress and neuroinflammation on the development of PD.

## 2. Oxidative Stress and PD

Oxidative stress, as one of the main culprits of DA neuron loss, has drawn special attention. The level of DA neurons is an important indicator to judge most neurological diseases, and PD is no exception. The massive loss of DA neurons will lead to the occurrence of PD [4]. ROS is mainly derived from mitochondria and considered to be natural by-products of oxygen metabolism under normal conditions [5]. On the one hand, ROS is an important physiological regulator of intracellular signaling pathways and plays an important role in maintaining homeostasis in the body. However, on the other hand, when the homeostasis is affected by endogenous or exogenous factors, ROS is rapidly and excessively produced, which is called oxidative stress [6]. Importantly, ROS is a messenger of certain signal transduction, which can activate some transcription factors and proteins, thus affecting different signal pathways and ultimately determining cell fate [7, 8]. In the brain of PD patients, the unstable DA could automatically oxidize to form quinone and H_2_O_2_, which react with iron or oxygen to form a more active OH^−^ [9]. DA quinone synthesized by catechin epoxidation of dopamine can interact with cysteine (Cys) residues to cause dopaminergic neurotoxicity. Since sulfhydryl groups on Cys are often located at the active site of functional proteins, the interaction between Cys residues and DA quinone may inactivate DA by removing sulfhydryl proteins, inhibiting protein functions, and interfering with mitochondrial functions. Researchers used dopamine-functionalized quantum dots (QDs) to monitor the interaction between DA quinone formation and subsequent Cys residues [10]. In addition, it has been proved that oxidation products of DA quinone are involved in brain mitochondrial dysfunction [11]. It is not difficult to judge that DA quinone does promote the pathogenesis of PD. Studies have found that proteins containing sulfhydryl groups easily form adducts with DA metabolites, suggesting that disrupting the intracellular protein thiol redox homeostasis would contribute to the development of PD [12]. The damage of oxidative stress to neurons is also manifested in lipid peroxidation, which leads to the structural damage of cell membrane and damage the integrity of cell membrane, thus generating cytotoxicity [13–15]. Targeted inhibition of lipid peroxidation prevented polymerization membrane interaction, abolished abnormal calcium flux, and restored physiological calcium signal. The new mechanism of cell death caused by lipid peroxidation was emphasized [16]. The correlation between oxidative stress and PD is also reflected in the changes of familial risk factors for PD, such *α*-syn, PINK1 (pten-induced kinase 1), Parkin (E3 ubiquitin ligase PARK2), DJ-1 (PARK7), and LRRK2 (leucine-rich repeat kinase 2). *α*-syn is a highly soluble unfolded protein that has been identified as the main component of Lewy bodies. The mutation of the SNCA gene encoded by *α*-syn is related to the occurrence of familial PD. The oxidative binding of dopamine to *α*-syn inhibits *α*-syn transition from fibrils to mature fibrils, resulting in potential cytotoxicity and accumulation of soluble fibrils in DA neurons [17]. Parkin, PINK1, DJ-1, and LRRK2 are proteins involved in autosomal recessive parkinsonism. In recent years, many studies have conducted in-depth exploration of the pathogenesis of PD by knocking out the above proteins [18]. Studies have shown that Parkin and PINK1 worked in the same biochemical pathway, targeting dysfunctional mitochondria for degradation, thus protecting the body from oxidative stress and inflammation [19]. DJ-1 is also confirmed to produce antioxidant properties [20]. Point mutations associated with PD in LRRK2 are associated with increased kinase activity [21].

In addition, aging leads to the accumulation of neuromelanin (NM), glutathione (GSH) depletion, and overloading of metal ions. All of the above substances are related to oxidative stress. NM is a product of the oxidative metabolism of DA and gradually accumulates with age. Since neurons could not degrade or eliminate NM, they will eventually occupy most of the cytoplasm of neurons. Human studies have shown that in PD patients, neuromelanin-forming DA neurons lose more than non-neuromelanin-forming DA neurons, suggesting that NM may affect the selective sensitivity of dopaminergic subgroups in PD patients [22, 23]. Studies in human SH-SY5Y neuroblastoma cells have found that NM can bind to neurotoxin HAAs (heterocyclic aromatic amines), making DA neurons that transmit neuromelanin more sensitive to HAA-induced neurotoxicity [24]. To break through the limitation of experimental animals such as rodents lacking NM, Vila et al. established the first rodent model to be tested in vivo. This model shows the generation and accumulation of age-dependent humanoid NM in dopaminergic melanotic neurons vulnerable to PD, which can reach the level of the elderly [25]. NM is a polymer composed of 5,6-dihydroxyindole (DHI), 5,6-dihydroxyindole-2-carboxylic acid (DHICA), benzothiazine unit, and lipid and protein. The presence of potential metal-binding sites in NM polymers includes catechol, carboxylic acid, and benzothiazide functional groups. X-ray absorption and infrared spectroscopy confirm that the iron in NM is coordinated by the dihydroxy catechol groups of DHI and DHICA [26]. LUHMES (Lund's human mesencephalic cells) were exposed to controlled materials from fe-nm-SIM and 3D silk fibroin scaffolds for 2 weeks, in which the precipitation produced peroxides that deplete nutrients/antioxidants, leading to cellular dysfunction [27]. In addition to its ability to interact with neuromelatonin and affect neuron cells, Fe has recently been found to have a typical form of cell death dependent on iron called ferroptosis, which is characterized by the accumulation of lipid peroxidation products and ROS [28]. The aggregation-membrane interaction is the key to induce ferroptosis, which depends not only on the conformation structure of the aggregates but also on the oxidation state of the lipid membrane. Inhibition of lipid peroxidation, reduction of iron-dependent free radical accumulation, and further prevention of neuronal oligomer-induced toxicity emphasize the role of increased ferritin in PD [16]. Studies have shown that ferroptosis induced by ferric ammonium citrate (FAC) may lead to apoptosis of MES 23.5 cells, and in the pathological process of PD mice, ferritin increased earlier than apoptosis [28]. Using a multimodal atlas of adult brain containing transcranial ultrasound (TCS) and quantitative susceptibility mapping, colocalization of hyperechoic substantia nigra and iron ion accumulation in PD was discerned [29]. In human neuroblastoma cell line SH-SY5Y, cobalt (Co) nanoparticles (NPs) and Co salts induce dose-dependent cytotoxicity, intracellular calcium increase, lipid peroxidation, and GSH depletion and trigger ferroptosis-like cell death [30]. Using the calculation tool INSIdE NANO, Co NPs have also been shown to have a potential connection with PD [31]. GSH is one of the most abundant thiol antioxidants in cells. As a key antioxidant, GSH gradually loses activity with age and accelerates oxidative stress. To solve the problem of bioavailability and quantification of GSH concentration in the CNS, a noninvasive nasal glutathione filling strategy was adopted to treat 15 patients with PD in the middle stage with a single dose of GSH nasal spray. The results of magnetic resonance spectroscopy (MRS) showed that the amount of GSH in the brain was significantly increased compared with that before administration [32]. It is suggested that nasal administration of GSH could increase its level in the brain of PD patients.

In summary, the imbalance between ROS production and consumption in the brain leads to oxidative stress, which in turn intensifies the accumulation of ROS. The consumption of antioxidant GSH further exacerbates the peroxidation state in the brain, placing DA neurons in an unstable state, making DA more vulnerable to the invasion of external metal ions. Eventually, DA automatically oxidize to form quinone and by-product neuromelanin, accelerating the development of PD. Oxidative stress also affects the PD process by regulating PD-related genes.

## 3. Neuroinflammation and PD

Neuroinflammation is a basic immune response of cells in the brain. Physiological concentration of inflammation protects neurons from damage, but excessive neuroinflammation exacerbates neuronal damage. Chronic neuroinflammation is one of the hallmarks of PD pathophysiology [33]. The neuroinflammatory response is regulated by immunocyte (such as microglia, astrocytes, and peripheral immune cells), cytokines, and chemokines. Microglia are the resident immune cells of the CNS. Under normal circumstances, the resting microglia play a pivotal role in maintaining tissue homeostasis and promoting brain development [34]. Upon activation by a variety of pathological stimuli, including infection, brain damage, stroke, and neurodegeneration, microglia secrete high levels of proinflammatory factors and the overaccumulation of these factors causes neuronal damage [35]. Recently, the intracellular inflammatory body complex, especially the NLRP3 inflammasome complex, is verified to be involved in the recognition and execution of host inflammatory responses [36]. Inflammasomes are a group of cytoplasmic multiprotein complexes that can identify a large number of stimuli through danger-associated molecular patterns (DAMPs) and pathogen-associated molecular patterns (PAMPs) [37]. Once the inflammasome sensor molecules are activated by triggers, they will undergo a conformational change, resulting in the loss of self-inhibitory state and oligomerization, thus becoming a platform for caspase-1 activation and inflammatory cytokine interleukin-1*β* (IL-1*β*) maturation. The activation of NLRP3 is not the result of a single cellular event, but a combination of multiple cellular events [38]. In a PD mouse model treated with the neurotoxin 1-methyl-4-phenyl-1,2,3,6-tetrahydropyridine (MPTP), compared with wild-type mice, the progression of PD in NLRP3-/- mice is suppressed, which also illustrates the connection between NLRP3 inflammatory bodies and PD [39]. Moreover, researchers have found that the downregulation of glial maturation factor (GMF) might regulate the cytotoxic functions of microglia and astrocytes by inhibiting the activation of NLRP3 inflammasome [40], which can play a beneficial role in preventing PD. Furthermore, the pharmacological inhibition of histone deacetylase 6 (HDAC6) by tubastatin A (TBA) could reduce NLRP3 inflammasome activation and protect DA neurons by peroxiredoxin 2 (Prx2) acetylation [41], thereby improving the PD process. In addition, mutation of genes associated with familial PD, such as Parkin, PINK1, and DJ-1, also causes neuroinflammation [42]. Some studies have reported abnormal inflammatory responses to LRRK2 in vivo. Researchers have reported that in mice mediated by recombinant adeno-associated virus vector (rAAV), the activation of microglia in the SN of LRRK2 transgenic rats with G2019S mutation was increased; this increase in neuroinflammation is accompanied by more pronounced neurodegenerative changes, which can be eliminated by inhibiting the activity of LRRK2 kinase [43]. Recently, another study showed increased expression of CD68 in G2019S LRRK2 microglia and increased expression of proinflammatory markers such as IL-6, TNF, and C1qa and astrocyte markers such as Vim, CD44, and Cxcl10 [44]. To sum up, LRRK2 is considered to be a proinflammatory factor in different animal models of neuroinflammation, and the increased activity of LRRK2 kinase is the driving factor of inflammation. In addition, studies have found that the absence of PINK1, a mitochondrial kinase associated with recessive familial PD, leads to congenital immune glial cell specific abnormalities. Specifically, the absence of PINK1 causes LPS/IFN-*γ*-stimulated inflammatory phenotype glial cells to secrete proinflammatory factors such as nitric oxide (NO), TNF-*α*, and IL-1*β* [45]. Parkin is another PD-related gene. In Parkin-knockout mice, striatum astrocytes are increased, and midbrain microglia are abnormally activated [46]. Moreover, after LPS stimulates microglial cells without Parkin, it expresses higher levels of proinflammatory cytokines, such as TNF-*α*, IL-6, and iNOS [47]; this suggests that Parkin plays an important role in the regulation of PD-associated inflammation. DJ-1-knockout mouse astrocytes produced higher levels of cyclocyte-2 (COX2) and IL-6 after LPS stimulation [48]. It suggests that DJ-1 deficiency may be involved in the pathogenesis of PD through astrocyte-mediated neuroinflammatory response.

Peripheral inflammation is also one of the sources of neuroinflammation, although the presence of the blood-brain barrier (BBB) excludes most components of the immune system [49]. However, this situation is not immutable. There is a risk of BBB destruction, which is recognized as a key factor in the development of neurological disorders. In fact, inflammation of the peripheral immune system has been reported to cause the occurrence and development of neurodegenerative diseases, such as PD, partly due to lymphocyte infiltration [33]. Abnormal immune responses were discovered during the development of PD, including changes in lymphocyte subsets in the cerebrospinal fluid and peripheral blood and increases in immunoglobulin synthesis, cytokines, and acute phase proteins [50]. The ratio of peripheral blood lymphocytes and regulatory T (Treg)/helper T 17 (Th17) lymphocytes in PD patients and the control group was analyzed by flow cytometry. The results showed that the percentage of NK cells (nonspecific cytotoxic lymphocytes) in patients with advanced PD was higher than that in the control group, while the percentage of CD3+ T cells was lower than that in the control group. The percentage of CD19+ B cells in male patients is lower than that in female patients, while NK cells increase, suggesting that peripheral blood lymphocyte subsets may be used as a marker of Parkinson's disease in the Chinese population [50]. In patients with PD, the T cell population changes and invades the CNS, causing neuronal degeneration and disease progression. T lymphocytes are an important part of adaptive immunity and cooperate with B cells to produce an immune response. Numerous studies have shown that microglia can be activated by chronic infiltration of surrounding inflammatory T cells [51, 52]. In the postmortem analysis of PD brain, infiltration of lymphocyte subtypes CD4+ and CD8+ cells was observed [53]. Moreover, altered intestinal environmental conditions are a classic example of a source of neuroinflammation. There is a growing recognition that a strong link between the condition of the gut and the functioning of the CNS exists [54]. This so-called gut-brain axis contains two-way communication between the central, enteric, and endocrine system, as well as the regulation of the immune response of the intestine and brain. All aspects of this system are severely affected by gut microbial activity [55, 56]. Stimulation in the intestine could trigger afferent signals of the vagus nerve. It is an important part of the neuroimmunoinflammatory reflex circuit and helps to enhance peripheral immune regulation. Studies have followed up 423 newly diagnosed PD patients for up to 5 years and found that the occurrence of gastrointestinal symptoms might be an early signature of cognitive impairment in PD [57].

To sum up, changes in the intestinal environment and infiltration of peripheral inflammatory factors lead to excessive neuroinflammation in the brain, further activating NLRP3 inflammasome and secreting more inflammatory factors. Such inflammatory environment exacerbates DA neuron damage and regulates the pathogenesis of PD through PD-related genes.

## 4. Crosstalk between Oxidative Stress and Neuroinflammation in the Progression of PD

### 4.1. Mitochondrial Dysfunction

Mitochondria play a key role in the function and survival of neurons in the brain. Mitochondria are dynamic organelles that continuously undergo fission and fusion, which are essential for maintaining mitochondrial function and homeostasis [58]. To keep the percentage of mitochondria with defective components to a minimum, mitochondrial fusion allows the exchange of mitochondrial contents, such as lipid membranes and mitochondrial DNA (mtDNA) [59]. As the main source of energy production and endogenous ROS production, mitochondria generate a series of consequences if their function is damaged. This mitochondrial dysfunction is due to the destruction of the balance of ROS accumulation and utilization in cells and tissues, the decrease of mitochondrial biology, the change of membrane potential, the decrease of mitochondrial number, and the change of oxidative protein activity [60]. Among them, the abnormal production of ROS is the primary factor that causes oxidative stress [61]. There is a lot of evidence that rotenone (ROT) is one of the risk factors for increased PD [62, 63]. Mechanically, ROT causes dysfunction of mitochondria in DA neurons and further disrupts the microtubule transport of neurotransmitter vesicles [64, 65]. ROT was used to treat PC12 cells, and flow cytometry with DCFH-DA fluorescent probe was used to evaluate the production of ROS in cells. ROS levels in ROT-treated cells were found to be significantly higher than those in the control group [66]. It is suggested that dysfunction of mitochondria in DA neurons will increase ROS levels. To explore the profound role of mitochondrial dysfunction on oxidative stress, the changes of mtDNA should be considered. First, mitochondria have a semiautonomous genome represented by mtDNA, which encodes certain structural components of the respiratory chain required to produce ATP, as well as the mechanism required for mitochondrial protein synthesis [67]. The half-life of mtDNA is relatively short, and the genes encoded in mtDNA have few noncoding sequences with each other. Coupled with continuous exposure to ROS, limited DNA repair mechanisms, and lack of histone protection, mtDNA is particularly vulnerable to oxidative damage, which finally leads to harmful mutations, including large-scale mtDNA rearrangements and point mutations [68]. Therefore, these accumulated mtDNA mutations might result in a decrease in the efficiency of electron transport chain (ETC), decrease in the production of ATP, and increase in the production of ROS [69]. In turn, the increase of ROS may cause the accumulation of more mutated mtDNA and form a positive feedback loop that increases mutations and ROS production, eventually leading to cell death. A great number of studies have shown that there is a good correlation between aging, accumulation of mtDNA mutations, decreased mitochondrial function, and increased oxidative stress during human and animal aging [68, 70].

It is worth noting that neuroinflammation did not stand idly by in this series of processes, but actively participated in it. The inflammatory response is induced by defective mitochondria, one of which is the release of mitochondrial contents into the cytoplasm or extracellular environment [71]. Under certain stress conditions, the outer and inner membranes of the mitochondria are damaged, and the release of mitochondrial components is induced. These mitochondrial components are recognized by pattern recognition receptors (PRRs) as DAMPs, indicating that cells are damaged, which trigger the innate immune response [72, 73]. The glycolytic enzyme hexokinase (HK) located in the outer mitochondrial membrane was identified as the PRR of n-acetylglucosamine. After the release of n-acetylglucosamine detected by HK, it separated from the mitochondrial outer membrane and released mtDNA, leading to the activation of NLRP3 inflammasome [74]. Studies have shown that human THP1 macrophages treated with mitochondrial complex 1 inhibitor ROT showed a dose-dependent increase in IL-1*β* secretion, accompanied by loss of mitochondrial membrane potential and ROS production. When NLRP3 was knocked out from THP1 cells, respiratory chain inhibitors did not induce IL-1*β* or caspase-1 secretion [75]. It suggests that mitochondrial dysfunction stimulates (chronic) inflammation through NLRP3 inflammasome-dependent inflammatory pathways. It was also reported that mitochondrial ROS, mtDNA, especially oxidized mtDNA mediate the activation of NLRP3 inflammatory bodies [76, 77]. The NLRP3 inflammasome complex acts as a sensor of mitochondrial dysfunction, and activation of this complex leads to the production of IL-1*β* [75]. Inflammatory cytokines can cause mitochondrial dysfunction and the production of ROS, leading to a self-toxic feedback loop. Moreover, studies have found that systemic injection of LPS induced regional specific expression of neuroinflammatory markers and altered mitochondrial activity and oxidative phosphorylation in normal mouse brain regions [78]. Combined with the close correlation between ATP levels and neuroinflammatory markers, it is believed that the underlying mechanism of acute neuroinflammatory response after systemic LPS injection might be through differential changes in oxidative stress, mitochondrial activity, and oxidative phosphorylation [78].

### 4.2. Glial Cells

The hallmark of neuroinflammation is microglia activation. As a type of glial cells found throughout the brain and spinal cord, microglia are the first and major form of CNS active immune defense [79]. Amounts of studies indicated that neuronal cell death in the substantia nigra of postmortem PD patients was accompanied by overactivation of microglia [80, 81]. Additionally, activated microglia exist in brain regions other than the substantia nigra, such as the pontine and basal ganglia [82], suggesting that microglia have a comprehensive role in the development of PD. One of the microglial cell PRRs, toll-like receptors (TLRs), is widely expressed in the CNS and plays different roles in neuron survival or death [83, 84]. It is a single-channel transmembrane protein, whose n-terminal extracellular ligand recognition domain carries leucine-rich repeats, while the c-terminal intracellular toll-like interleukin-1 receptor (TIR) domain converts extracellular recognition into an intracellular response [85]. The TIR domain interacts with adaptive molecules, such as MyD88, TRIF, and TRIF-related adaptor molecule (TRAM), in response to various stimuli [86]. In order to cope with immune challenges, pathogens, and injuries, branched microglia underwent a complex and multistage activation process, and their morphology rapidly changed into activated microglia cells, taking on the shape of amoeba. Interestingly, the activated microglia are composed of two groups of cells that have distinct or even opposite functions. These two extremes of microglial polarization are known as the classically activated M1 (proinflammatory) and alternately activated M2 (anti-inflammatory) phenotypes [87]. The morphologically altered M1 microglia have a phagocytic effect and release a large number of inflammatory factors, such as tumor necrosis factor (TNF-*α*), IL-6, IL-1, and nitric oxide (NO). The accumulation of these factors further leads to the loss of DA neurons in PD patients [88]. In contrast, M2 microglia secrete anti-inflammatory cytokines, such as IL-4, IL-13, IL-10, transforming growth factor (TGF), and neurotrophic insulin-like growth factor-1 (IGF-1), through phagocytic cell fragments or damaged neurons to reduce inflammatory response and accelerate repair [89]. It can be seen that microglia-mediated neuroinflammation plays a dual role in alleviating and aggravating the progression of PD [82]. In addition to being activated by neuroinflammation, microglia are major contributors to oxidative stress in the CNS [90]. It has been mentioned above that in the state of oxidative stress, ROS accumulate in large quantities, and ROS selectively modify proteins by targeting thiol functional groups on cysteine residues [91]. These reversible modifications can be likened to phosphorylation, which regulates protein function as an important signal transduction process in various cell types, including microglia. The production of ROS in microglia comes from a variety of sources, such as intracellular peroxidase, membrane surface NADPH oxidase (NOX), and mitochondrial oxidation. NOX is a kind of multi-subunit enzyme, which is involved in microglia-mediated neurotoxicity through two mechanisms. First, activation of NOX elicits the production of extracellular ROS. Second, the activation of NOX leads to the increase of intracellular ROS in microglia, which mediates the change of microglia morphology and increases the expression of proinflammatory genes [92]. Considering the dual effects of NOX activation on neurotoxicity and the ubiquity of NOX activation to microglia, it is suggested that NOX in microglia is a key mechanism of neuronal death in a variety of neurodegenerative diseases [93]. Moreover, microglia not only present proinflammatory and anti-inflammatory abilities under the action of neuroinflammation but also have sufficient antioxidant defense mechanisms [94]. The prevention of oxidative damage in mitochondria is crucial for microglia, and the mitochondrial GSH system plays an important role in the antioxidant potential of microglia mitochondria [95]. It is accounted by the high concentration of GSH and antioxidant enzyme Cu/ZnSOD in microglia to prevent oxidative and nitrosation damage and achieve the function of defense and repair of the brain [96]. Collectively, microglia activated by either neuroinflammation or oxidative stress can confer two opposite effects, which further proves the duality of microglia.

In addition to microglia, another subtype of glial cells is astrocytes, which are star-shaped, and a single human astrocyte can interact with up to 2 million neurons at a time [97]. Like microglia, astrocytes are essential for maintaining the health of neurons. They provide structural and metabolic support and regulate synaptic transmission, water transport, and blood flow in the brain. Also, they produce a variety of neurotrophic molecules, such as glial cell-derived neurotrophic factor (GDNF), which is particularly important for the development and survival of DA neurons [98, 99]. In addition, high levels of DJ-1 immunity were found in astrocytes surrounding idiopathic PD pathology [100]. DJ-1 is encoded by the familial PD-related gene PARK7 and has multiple functions, including transcriptional regulation, antioxidative stress response, and mitochondrial regulation. Its activity is regulated by its oxidation state, which reflects oxidative damage response. A pseudotype lentiviral gene transfer vector with specific tropism for CNS astrocytes has been developed, which can overexpress human DJ-1 protein in astrocytes [101]. Some researchers have found that overexpression of hDJ-1 in astrocytes of Lewis rats treated with ROT can protect them from the effects of ROT-induced neurodegeneration, and the oxidative stress of neurons and microglia cell activation are significantly reduced [101]. An interesting study found that an octadecaneuropeptide (ODN) associated with a diazepam binding inhibitor (DBI) synthesized and released by glial cells can protect neurons and astrocytes from oxidative stress-induced apoptosis through its transport receptor [102]. Relative to the A2 phenotype with neuron protection polarized under hypoxia, there is another subtype called A1, which is induced by classically activated neuroinflammatory microglia. Lipocalin-2 (LCN2) is thought to be a chemokine inducer and an autocrine promoter of the classical proinflammatory activation of astrocytes [103]. A1 astrocytes lose their ability to promote neuronal survival, growth, synaptic formation, and phagocytosis, resulting in neuronal and oligodendrocyte death [104]. Studies have confirmed that extracellular vesicles (EVs) derived from astrocytes can diffuse and amplify ethanol-induced neuroinflammation through TLR4 activation, thereby acting as cytokine for inflammatory signaling [105]. A dietary triterpene Lupeol was also proved to play a neuroprotective role by regulating the activation state of astrocytes and the expression of neurotrophic factors and inflammatory factors [106].

### 4.3. PD-Related Signaling Pathways

The NF-*κ*B transcription factor family is multidirectional and provides a mechanism for cells to respond to various inflammatory stimuli. NF-*κ*B is usually present in the cytoplasm as a heterodimer, p50/p65 (RelA) is the most abundant form, and the Rel homology domain (RHD) in these proteins is responsible for dimerization [107]. The activity of NF-*κ*B is mainly regulated by the interaction with the inhibitory I*κ*B protein. The interaction between I*κ*Ba and NF-*κ*B p50-RelA dimer is the most studied. This interaction blocks the ability of NF-*κ*B to bind to DNA. In most cells, NF-*κ*B exists in the cytoplasm in the form of a latent, inactive, I*κ*B-bound complex. However, the NF-*κ*B pathway can be activated through a variety of pathways. With activation by multiple extracellular signals (including ROS, TNF-*α*, IL-1*β*, and LPS), NF-*κ*B quickly enters the nucleus and activates gene expression [108]. In addition, studies have shown that neurotropin (NTP), a drug for the treatment of chronic pain and peripheral inflammation, inhibits the activation of NF-*κ*B signaling by reducing the transposition of p65 to the nucleus, thus decreasing the production of proinflammatory mediators and exerting anti-inflammatory effects [109]. At the same time, the cysteine residue (Cys-62) in p50 subunit is easily oxidized, suggesting that NF-*κ*B heterodimer could be directly modified under the conditions of increased oxidative stress. Interestingly, this domain exhibits spatial redox regulation, where it is oxidized in the cytoplasm but reduced in the nucleus. Activation of NF-*κ*B depends on the degradation of I*κ*B, and ROS can induce I*κ*B kinase (IKK) phosphorylation and degradation [110].

Nuclear factor erythroid 2-related factor 2 (Nrf2) is a key transcription factor that regulates cellular redox state in response to oxidative stress [111]. Nrf2 acts on the antioxidant response element (ARE) genes. Under normal circumstances, Nrf2 is anchored by Kelch-like ECH-associated protein 1 (Keap1) in the cytoplasm and degraded by the ubiquitin proteasome pathway [112]. Upon stimulation by an oxidative stress insult, Nrf2 is excreted from Keap1 and translocates into the nucleus and then binds to ARE to further activate the transcription of a wealth of antioxidant and cytoprotective genes [113], such as nicotinamide adenine dinucleotide phosphate:quinine oxidoreductase-1 (NQO1), hemeoxygenase-1 (HO-1), superoxide dismutase (SOD), and glutamate-cysteine ligase catalytic subunit (GCLC) [114]. Furtherly, Nrf2 is located in the cytoplasm of DA neurons of the substantia nigra of a healthy brain [115]. However, in the age-matched PD patients, Nrf2 is located in the nucleus and the expression of NQO1 and HO-1 in the Nrf2 signaling pathway is upregulated [116]. Therefore, the activation of Nrf2 signaling pathway holds a promising capacity to attenuate PD. In addition to its beneficial antioxidant effects, Nrf2 also plays an anti-inflammatory role. Emerging evidence suggests that after Nrf2 activation, the transcription of proinflammatory cytokines in microglia is inhibited [115]. Dimethyl fumarate (DMF), as an Nrf2 activator, mediates DA neuroprotection and inhibits the activation of microglia and astrocytes [117]. At the same time, studies have shown that Icariin (ICA) attenuated glial cell-mediated neuroinflammation and confer DA neuroprotection in an Nrf2-dependent manner [118], suggesting that the inhibition of neuroinflammation could be achieved through the activation of Nrf2. On the other hand, the activation mechanism of Nrf2 anti-inflammatory ability involves NF-*κ*B [115, 119]. For example, the NF-*κ*B binding site is in the promoter region of the Nrf2 gene, suggesting the interaction between two transcription factors [120]. Also, except for controlling redox activity, Nrf2 inhibits the upregulation of proinflammatory gene expression [121].

Signal transduction and transcriptional activator 3 (STAT3), a member of the STAT protein transcription factor family, has been widely described as a central signaling molecule that controls cellular adaptation to environmental stimuli or stress. In recent years, studies have gradually revealed the correlation between STAT3 and PD. As a regulatory factor for inflammatory gene expression, STAT3 plays an important regulatory role in the inflammatory mechanism of PD. For example, HSP70 regulates rotenone-induced inflammatory effects in PD models through the STAT3 pathway [122]. At the same time, STAT3 plays an important regulatory role in the response of microglia to various stimuli, mediating the proinflammatory response of microglia to various central nervous system injuries [123]. Activated STAT3 promotes the transcription and expression of many genes that encode proinflammatory mediators, including cytokines, chemokines, adhesion molecules, and inflammatory enzymes. Accumulating evidence demonstrated that STAT3 is involved in inflammation-related damage [124, 125]. In an LPS-induced neuroinflammatory model in vitro, the anti-inflammatory factor IL-10 protects ventral midbrain (VM) neurons from neuroinflammation by inducing phosphorylation of STAT3 [124]. Meanwhile, STAT3 could increase the expression of mitochondrial coding transcripts, enhance oxidative metabolism, and participate in the regulation of oxidative stress [126]. In turn, the destruction of activity (or inhibition) of the oxidative phosphorylation complex and changes in oxygen consumption rate led to the activation of mitochondrial STAT3 (mitoSTAT3) and abnormal respiratory function [127].

In general, through the description of mitochondrial dysfunction, activated glial cells and PD-related signaling pathways revealed the crosstalk effect of oxidative stress and neuroinflammation in PD pathogenesis through the above three aspects.

## 5. Conclusion

**Figure 1:**
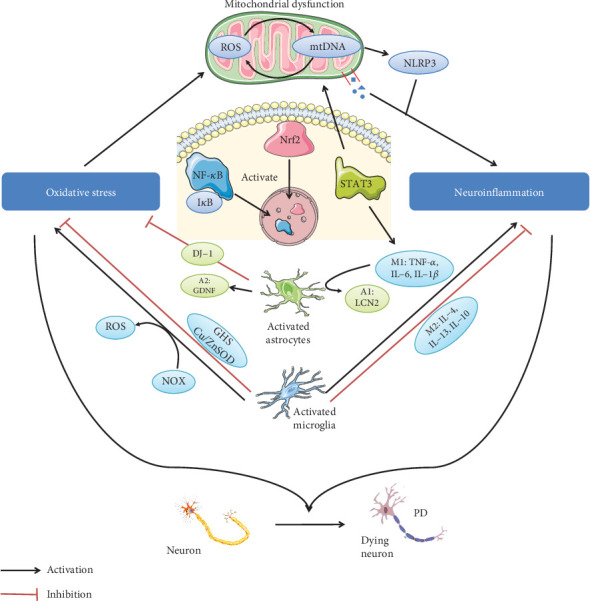
Crosstalk between oxidative stress and neuroinflammation in the progression of PD. Oxidative stress and neuroinflammation played synergistic roles in the development of PD. First, ROS increased during oxidative stress. Continuous exposure to ROS led to mtDNA mutation, and mtDNA mutation accumulation enhanced ROS production, forming a positive feedback loop. On the one hand, defective mitochondria released their contents to trigger an innate immune response. Next, oxidative mtDNA mediated the activation of NLRP3 inflammasome to generate cytokines to induce neuroinflammation. Moreover, there were three signaling pathways that played key roles in the PD process: NF-*κ*B, Nrf2, and STAT3. After the inhibitory protein I*κ*B that bound to NF-*κ*B was degraded by ROS, NF-*κ*B entered the nucleus and mediated inflammation. Nrf2 was also activated and entered the nucleus, which simultaneously generated anti-inflammatory and antioxidant properties. Activated STAT3 mediated the proinflammatory response of microglia to CNS injury, including promoting the secretion of inflammatory factors. Finally, there were resident glial cells in the brain: microglia and astrocytes. The former, as a marker of neuroinflammation, could be polarized to the M1/M2 phenotype with proinflammatory and anti-inflammatory effects. Moreover, microglia not only induced oxidative stress through NADPH oxidase- (NOX-) generated ROS but also prevented oxidative damage through high concentrations of GSH and Cu/ZnSOD. In addition, astrocyte-overexpressed DJ-1 exerted antioxidative stress and induced A1 astrocytes to polarize to A2 phenotype in hypoxic state to further produce GDNF to support neuron growth. Moreover, astrocytes were activated by microglia M1 phenotype to become astrocyte A1 phenotype, thus losing their protective functions. (The signals coming from microglia are contained in the light blue ellipse, while signals in the green ellipse are produced by astrocytes.)

Oxidative stress and neuroinflammation are an important entry point to study the pathogenesis of PD. Oxidative stress is the result of unbalanced production and consumption of ROS in the brain. Exogenous effects and changes in the internal environment make the brain vulnerable to oxidative stress. Neuroinflammation is formed by the accumulation of a large number of proinflammatory factors in the brain through BBB. The infiltration of lymphocytes and the influence of intestinal flora cannot be ignored (Table 1). Thus, the pathogenesis of PD is an extremely complex process, which is not generally caused by a single factor, and largely resulted from the synergistic effects of oxidative stress and neuroinflammation. In fact, studies have reported that chronic inflammation induced by LPS can cooperate with ROT-induced oxidative stress to aggravate the damage of DA neurons [128]. It is uneasy to discuss oxidative stress and neuroinflammation separately in the study of the pathogenesis of PD, because there is a crosstalk between oxidative stress and neuroinflammation (Figure 1). They are, respectively, mitochondrial dysfunction caused by oxidative stress and neuroinflammation induced by mitochondrial dysfunction. Glial cells (microglia and astrocytes) and PD-related signaling pathways (NF-*κ*B, Nrf2, and STAT3) can play a regulatory role in both neuroinflammation and oxidative stress.

Taken together, oxidative stress and neuroinflammation promote each other's development under the premise of performing their respective functions and play a synergistic role in inducing and aggravating PD. This provides a more comprehensive way of thinking for the subsequent research on the pathogenesis of PD and the development of therapeutic drugs. That is to say, the development of therapeutic drugs can focus on the interaction between oxidative stress and neuroinflammation, so as to get twice the result with half the effort in treatment. In this review, the synergistic effect of oxidative stress and neuroinflammation in the pathogenesis of PD has been described, but whether there is a relationship between incentives or facilitators needs further illumination.

## Figures and Tables

**Table 1:** Occurrence and development of oxidative stress and neuroinflammation associated with PD.

	Oxidative stress	Neuroinflammation
Occurrence	(+) ROS	Microglia activation
Development	Broken redox balance	(+) Inflammation cytokine
Aggravation	(+) Lipid, neuromelanin, metal ions (-) Glutathione	NLRP3 activation, lymphocyte infiltration, intestinal flora disorder
PD-associated genes	Parkin, PINK1, DJ-1, LRRK2	
Consequence	DA damage	
